# Portal vein gas in emergency surgery

**DOI:** 10.1186/1749-7922-3-21

**Published:** 2008-07-17

**Authors:** Abdulzahra Hussain, Hind Mahmood, Shamsi El-Hasani

**Affiliations:** 1 General surgery department, Princess Royal University Hospital, Greater London, UK

## Abstract

**Background:**

Portal vein gas is an ominous radiological sign, which indicates a serious gastrointestinal problem in the majority of patients. Many causes have been identified and the most important was bowel ischemia and mesenteric vascular accident. The presentation of patients is varied and the diagnosis of the underlying problem depends mainly on the radiological findings and clinical signs. The aim of this article is to show the clinical importance of portal vein gas and its management in emergency surgery.

**Methods:**

A computerised search was made of the Medline for publications discussing portal vein gas through March 2008. Sixty articles were identified and selected for this review because of their relevance. These articles cover a period from 1975–2008.

**Results:**

Two hundreds and seventy-five patients with gas in the portal venous system were reported. The commonest cause for portal vein gas was bowel ischemia and mesenteric vascular pathology (61.44%). This was followed by inflammation of the gastrointestinal tract (16.26%), obstruction and dilatation (9.03%), sepsis (6.6%), iatrogenic injury and trauma (3.01%) and cancer (1.8%). Idiopathic portal vein gas was also reported (1.8%).

**Conclusion:**

Portal vein gas is a diagnostic sign, which indicates a serious intra-abdominal pathology requiring emergency surgery in the majority of patients. Portal vein gas due to simple and benign cause can be treated conservatively. Correlation between clinical and diagnostic findings is important to set the management plan.

## Background

Portal vein gas (PVG) represents a challenge for diagnosis and management of the underlying surgical cause in emergency surgery. PVG is not a disease; it is a diagnostic clue in patients who may be harbouring an intra-abdominal catastrophe [1]. Mortality could reach 75% [2], however, an increasing number of cases associated with benign conditions suitable for conservative treatment are being reported [3,4]. The majority of patients present acutely to the Accident and Emergency department. Following assessment, surgeons will be alarmed immediately by the patients' critical and unstable condition. A significant number of patients with PVG are misdiagnosed and admitted to the medical ward because they are elderly and unwell and are subsequently not subjected to the specific diagnostic tools. The most common underlying pathology includes bowel ischemia, alteration of the gastrointestinal lining, inflammation and sepsis. However, PVG has recently been recognized as a rare complication of endoscopic and radiological procedures [5].

## Methods

A computerised search was made of the Medline for publications discussing portal vein gas through March 2008. The search words were 'hepatic portal vein gas',' portal vein gas'. Other articles were identified by cross referencing. Sixty articles were identified and selected for this review because of their relevance. These articles cover a period from 1975–2008. The main topics were the aetiology of PVG, its presentation, investigations and the management. The conclusions of the major studies included in this review are presented and constitute the current concept of the significance of PVG in emergency surgery.

## Results

Two hundreds and seventy-five patients with gas in the portal venous system were reported. The commonest cause for portal vein gas was bowel ischemia and mesenteric vascular pathology (61.44%). This was followed by inflammation of the gastrointestinal tract (16.26%), obstruction and dilatation (9.03%), sepsis (6.6%), iatrogenic injury and trauma (3.01%) and cancer (1.8%). Idiopathic portal vein gas was also reported in1.8 %. Men and women affected equally. Majority of the patients are elderly and affected by most serious cause, the mesenteric vascular pathology. Diagnostic approach is based on the clinical suspicion and investigations such as CT scan which represents the standard test. The management depends on the underlying cause. Better outcome is reported because of advances in diagnostic facilities, the intensive care setting and less invasive operative technique such as laparoscopy.

### Aetiology

**Figure 1:**
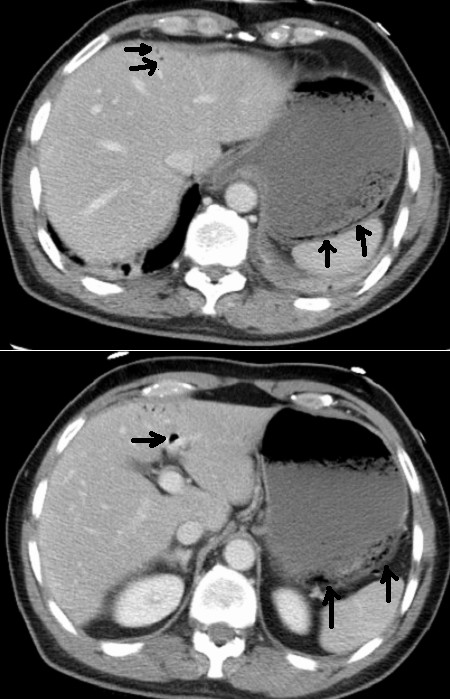
Portal gas within the liver secondary to partial necrosis of the gastric wall.

**Figure 2:**
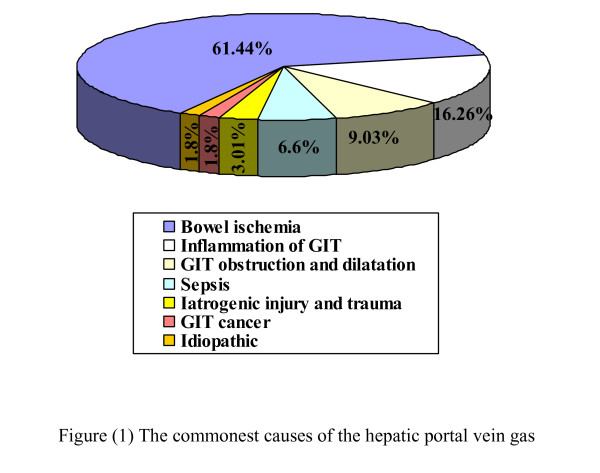
Commonest causes of portal vein gas.

The most important cause for PVG is bowel ischemia, inflammation of the gastrointestinal tract GIT (see figure 1), obstruction and dilatation, gastro-intestinal leak, infection, diffuse peritonitis and sepsis [6-17] (see figure 2). It may follow GIT cancer, gastric ulcer, hypertrophic pyloric stenosis, cystic fibrosis and acute appendicitis [18-21]. PVG is also reported following perforated and non perforated diverticulitis, oesophageal and colonic carcinoma [22-26]. Inflammation of the GIT such as necrotizing enterocolitis, Crohn's disease and ulcerative colitis [27-30] are also reported in literatures. It is also reported following surgical procedures such as pancreaticoduodenectomy [31]. Vascular causes include septic thrombophlebitis of the superior mesenteric vein, superior mesenteric artery syndrome, and hemodialysis [32-35].

Respiratory tract infection, abdominal trauma, diagnostic and therapeutic gastrointestinal procedures, resuscitation procedures, drugs and caustics ingestion are rare causes for PVG, while it is idiopathic in certain cases [36-44] (see additional file 1).

### Presentation

Portal vein gas is a radiological sign of underlying pathology mostly related to the gastrointestinal tract [5]. The presentation is that of primary disease and in the majority of patients is characterised by acute onset of abdominal pain and carries relatively high morbidity and mortality despite modern surgical and medical management. Other patients are asymptomatic and PVG is discovered during investigation for another problem. No special predilection for male or female and PVG usually affect elderly patients, although cases affecting the younger population including infants have been reported as well [18].

### Diagnosis

#### Clinical findings

An accurate history and thorough examination are crucial for the management plan. Correlation between the clinical signs and the radiological findings will be the most important factor in the decision to proceed to laparotomy or not. Acute abdominal signs and positive radiology will mandate emergency operation in the majority of cases. On the other hand positive radiology with no acute abdominal signs may indicate a conservative treatment especially in elderly frail patients.

#### Computed axial tomography CT

Is superior to other radiological modalities and it is the gold standard for diagnosis of PVG and its aetiology [45,46] (see figure 2). At CT, portal vein gas appears as tubular areas of decreased attenuation in the liver, predominantly in the left lobe [47]. The extent of PVG depends on the cause and severity of the underlying pathology and therefore the demonstration of a small amount of gas in the portal vein may indicate a less severe condition and suggest a less invasive approach. [48]. In these cases CT scan can provide crucial information for diagnosis and whether emergency surgery is needed or not. The most important information provided by CT scan is to rule out bowel ischemia, thus a negative CT scan could help to decide the next step of management which could be a conservative one. Although modern imaging contributes to the safe and effective management plan, CT scan however, may be of limited value in certain cases where the prediction of the extent of the gastrointestinal ischemia can not be accurately assessed. Therefore partial thickness infarction and necrosis which can produce PVG and pneumatosis intestinalis may be undetectable by CT study [49].

#### Ultrasound examination US

Can be used to assess the PVG and predict the outcome[50]. The ultrasound features include dot-like, streak-like and fruit-pulp-like patterns [51]. The real time phase of US is advantageous to detect the PVG and may be of comparable accuracy to the CT scan, however CT scan is more valuable in detecting the aetiology of PVG or excluding abdominal catastrophes [52]. When US is positive, urgent clinical evaluation followed by contrast-enhanced CT is indicated to assess for associated intestinal ischemia[53].

#### Colour Doppler flow imaging (CDFI)

The efficacy of this modality is less than CT scan in detection of PVG [54].

#### Magnetic resonance imaging MRI

This modality will delineate the portal system and is helpful in the assessment of patency of the portal vein and associated tributaries and whether there is occlusive or non occlusive ischemia.

#### Plain abdominal radiology

The plain film is of no specific diagnostic criteria; nevertheless, it can show indirect signs such as oedematous bowel wall, ileus, gasless abdomen, or gas in the bowel wall and portal vein in more severe cases of bowel ischemia. Plain abdominal x-ray can detect PVG in about 12.5% of cases [55].

#### Blood test

An increase in the white cell count in more than 50% of cases and lactic acidosis in more than 90% of patients with established bowel ischemia is usually confirmed. Polycythemia and thrombocythemia may indicate a clue to the likely cause. These non specific parameters will help in the overall assessment of patients.

#### Management

Is largely applied to the underlying cause (see Additional file 1). However, there are cases where a surgeon will proceed to abdominal exploration in the presence of borderline abdominal signs. Gas in portal vein is a good predictive factor for diagnosis, management, and prognosis [56]. In the past the traditional teaching is to operate once you have radiological evidence of PVG. However with the advances in diagnostic radiology, endoscopy, baseline support and intensive care setting, a less invasive approach is suggested especially for benign cases which indicate no intrabdominal catastrophe and thus reducing the rate of negative laparotomy [57]. Endoscopy can be very helpful in assessment or exclusion of upper and lower gastrointestinal pathology in the presence of PVG when CT scan gave no clue to the diagnosis, therefore a more sensible decision can be made. Patients diagnosed by CT scan with obstruction, infarction or vascular problems need no further endoscopic confirmation and are candidates for emergency exploration.

Therefore, patients who present with PVG can be divided into three categories: Firstly: those who have acute abdominal features and positive diagnostic criteria will need immediate exploration by laparoscopy or laparotomy.

Secondly: Patients who have positive diagnosis of PVG but without abdominal signs, are relatively stable and have no mesenteric ischemia will be good candidates for a conservative approach [58].

Thirdly: Patients who have borderline features of acute abdomen and less dense diagnostic signs of PVG may need endoscopic examination of the upper and lower GIT as a safe approach rather than to wait for underlying bowel ischemia [59]. This can be achieved using diagnostic laparoscopy which can help identify GIT pathology. In non GIT cause such as vascular disease or when the problem cannot be managed laparoscopically, laparotomy is indicated.

The history, severity of clinical signs and the density of PVG depicted by diagnostic tests will direct the management to either emergency intervention or conservative plan. With prompt surgical response a high mortality figure of 86% in bowel ischemia can be reduced to an acceptable figure [60]. Although the amount of gas is correlated with the severity of the pathology, the prognosis however, was related to the pathology itself and was not influenced by the presence of the gas in the portal system [6].

## Conclusion

Portal vein gas is a diagnostic sign which indicates a serious intra-abdominal pathology requiring emergency surgery in the majority of patients. Benign hepatic portal vein gas due to less dangerous disease can be treated conservatively after exclusion of intra-abdominal catastrophe. Correlation between clinical and diagnostic findings is important to set the management plan.

## Abbreviations

PVG: Portal vein gas; GIT: Gastrointestinal tract; CT: Computarised axial tomography; US: Ultrasound.

## Competing interests

The authors declare that they have no competing interests.

## Authors' contributions

AH carried out the Medline search and participated in the sequence alignment and drafted the manuscript. HM carried out the statistical data and formatted the figures and tables. SEL conceived the study, and participated in its design and coordination and helped to draft the manuscript. All authors read and approved the final manuscript.

## Supplementary Material

Additional file 1 Clinical conditions associated with portal vein gas. Click here for file
