# Supplementary material for: Portal vein gas in emergency surgery
Source: World J Emerg Surg. 2008 Jul 17;3:21. doi: 10.1186/1749-7922-3-21 (PMC2490689; doi:10.1186/1749-7922-3-21)
Supplement: Additional file 1 — Clinical conditions associated with portal vein gas. [file 1749-7922-3-21-S1.doc]

| Table(1) Clinical conditions associated with portal vein gas | |
| --- | --- |
| Gastrointestinal cause | Non gastrointestinal cause |
| Gastric ulcer  Gastric volvolus  Acute gastric dilatation  Emphysematous cholecystitis  Cholangitis  Bowel ischemia  Bowel obstruction  Bowel necrosis  Distended but non-necrotic bowel  Severe gastroenteritis  Terminal ileitis  Perforated diverticulitis  Non complicated diverticulitis  Acute appendicitis  GIT cancer (gastric,esophageal , colonic carcinoma)  Necrotizing enterocolitis  Hypertrophic pyloric stenosis Ulcerative colitis  Crohn's disease  Abdominal trauma  Acute pancreatitis  Percutaneous endoscopic gastrostomy tube placement Gastrointestinal anastomotic leak  Blunt colon injury  Following diagnostic and therapeutic procedures of the gut ,eg ERCP and sphincterotomy, gastric biopsy  Pancreaticoduodenectomy | Peritonitis  Intra-abdominal abscess  Septic thrombophlebitis of the superior mesenteric vein  Superior mesenteric artery syndrome  Enterovascular fistula  Long-term hemodialysis  Prolonged cardiopulmonary resuscitation CPR  Drugs (irinotecan and cisplatin, colchicine toxicity,  Ileus due to anticholinergic medication  Ingestion of a caustic substance and hydrogen peroxide  Bronchopneumonia  Barium enema examination  Out-of-hospital cardiac arrest  Idiopathic  Graft-vs-host reaction  Viral infection  After transplantation  Cystic fibrosis |
